# Development of *Bacillus subtilis* mutants to produce tryptophan in pigs

**DOI:** 10.1007/s10529-016-2245-6

**Published:** 2016-11-03

**Authors:** Karin Bjerre, Mette D. Cantor, Jan V. Nørgaard, Hanne D. Poulsen, Karoline Blaabjerg, Nuria Canibe, Bent B. Jensen, Birgitte Stuer-Lauridsen, Bea Nielsen, Patrick M. F. Derkx

**Affiliations:** 1 Chr. Hansen A/S, Bøge Allé 10-12, 2970 Hørsholm, Denmark; 2 Department of Animal Science, Aarhus University, Foulum, 8830 Tjele, Denmark

**Keywords:** *Bacillus subtilis*, mtrB, trpS, Tryptophan, UV-mutagenesis

## Abstract

**Objectives:**

To generate tryptophan-overproducing *Bacillus subtilis* strains for in situ use in pigs, to reduce the feed cost for farmers and nitrogen pollution.

**Results:**

A novel concept has been investigated—to generate *B. subtilis* strains able to produce tryptophan (Trp) in situ in pigs. Mutagenesis by UV was combined with selection on Trp and purine analogues in an iterative process. Two mutants from different wild types were obtained, mutant 1 (M1) produced 1 mg Trp/l and mutant 2 (M2) 14 mg Trp/l. Genome sequence analysis revealed that M1 had three single nuclear polymorphisms (SNPs) and M2 had two SNPs compared to the wild type strains. In both mutants SNPs were found in genes regulating tryptophan synthesis. Reverse transcription PCR confirmed up-regulation of the tryptophan synthesis genes in both mutants, the expression was up to 3 times higher in M2 than in M1.

**Conclusions:**

Tryptophan-excreting *B. subtilis* strains were obtained with UV-mutagenesis and analogue selection and can be used in animal feed applications.

**Electronic supplementary material:**

The online version of this article (doi:10.1007/s10529-016-2245-6) contains supplementary material, which is available to authorized users.

## Introduction

Bacilli are used extensively in the biotechnology industry to produce a vast range of enzymes (van Dijl and Hecker 2013) as well as probiotics in animal feed (Cutting 2011). Their use in the feed industry is related to their probiotic effect including improved intestinal health and growth performance of pigs and poultry, thereby reducing or replacing the use of antibiotics. Due to increasing prices of e.g. soy bean or fishmeal, alternative protein sources with a lower protein value and insufficient amounts of essential amino acids are being utilized for pig feed. To supply the essential amino acids required for the animals, diets with high dietary crude protein content are formulated, leading to both ineffective growth of the pigs and heavy environmental nitrogen load. A typical pig feed ration in Europe results in a nitrogen excretion of approx. 55% (w/w) of the ingested nitrogen (Poulsen et al. 2013). A higher provision of the limiting essential amino acids while keeping the dietary crude protein level low would result in reduced nitrogen loss to the environment and improved production efficiency (Nørgaard et al. 2014).

At present, there are five L-amino acids available for use in pig feed formulation: lysine, threonine, methionine, tryptophan (Trp), and valine (Val). Industrial production of these amino acids is by fermentation, often using genetically-modified organisms (Ikeda 2006). Due to legislative restrictions in the food and feed industry the use of recombinant DNA technology is highly controversial, thus natural methods such as UV-mutagenesis or chemical treatment have to be applied to obtain amino acid-producing strains for use as probiotics. These methods are approved by authorities and has consumer acceptance, and have been extensively used in the food industry (Derkx et al. 2014).

By additional selection using amino acid analogues, such as 5-fluoro-dl-tryptophan (5-FT), a structural Trp analogue, *Bacillus* strains producing up to 13.6 g Trp/l have been reported (Kurahashi et al. 1987).

As many *Bacillus* species are generally regarded as safe the use of cells or spores from a naturally improved strain, delivering amino acids in situ would bring added value to the farmers.

## Materials and methods

### Strains

The *Bacillus subtilis* strains used in the present study are summarized in Table 1.

**Table 1:** *Bacillus subtilis* strains used in the current study

Strain	Relevant feature (s)	CHCC number^a^
WT1		CHCC26701
WT2		CHCC26702
M1	Trp producing strain, mutant of WT1. Three SNPs compared to the WT1 strain	CHCC26703
M2	Trp producing strain, mutant of WT2. Two SNPs compared to the WT2 strain	CHCC26704

^a^Chr Hansen culture collection

### Media and culture conditions

Veal Infusion Broth (VIB, Difco) was used for propagation by inoculating and incubating overnight at 37 °C and 150 rpm. Chemically defined media (CDM1 and CDM2) were used for different assays [Supplementary Table 1; (Kurahashi et al. 1985; Leitch and Collier 1996)]. Chemical-defined medium 1 agar was used for the dominant selection on Trp analogues; CDM1 or CDM2 were used for assaying Trp, and CDM2 for shake-flask growth assays. For growth assays cultures were propagated overnight at 37 °C and 150 rpm, in 6 ml lysogeny broth.

### Analogues and isolation of resistant mutants

Two structural Trp analogues and a purine analogue were used for strain development: 5-fluoro-dl-tryptophan (5-FT) from 500 to 5000 µg/ml and 8-aza-guanine (8-AZA) from 5 to 200 µg/ml. The mutants were obtained after iterative rounds of UV mutagenesis and growth on the different analogues in different concentrations (Supplementary Fig. 1, Additional methods 1). For testing Trp production, cultures were inoculated with 1% (v/v) in 6 ml CDM2, and grown at 37 °C and 150 rpm. At 24 and 48 h, samples were taken for OD_600_ measurements and amino acid analysis.

### Growth and tryptophan production in shake flasks

Overnight cultures were inoculated into 75 ml CDM2 in a 500 ml shake-flask to a start OD of 0.1 and incubated at 37 °C and 150 rpm. Samples for cell dry weight (CDW) and Trp measurements were taken throughout incubation (24 h). Cell dry weight was measured by filtering culture through a 0.22 μm membrane filter and drying in a microwave at 350 W for 8 min. Samples for Trp measurements were filtered through a 0.2 μm filter and stored at −20 °C until analysis. The growth experiments were performed in biological duplicates, with less than 10% deviation between the cultures.

### Sequencing and analyzing genomic DNA, single nucleotide polymorphism analysis

Genomic DNA was prepared from the four *B. subtilis* strains with the DNeasy Blood and Tissue kit (Qiagen GmbH). DNA was sent for sequencing at Beijing Genomics Institute, using an Illumina HiSeq platform. The sequencing data has been saved as BioProject SRR3211037.

The genome sequences were analyzed using the CLC Genomics Workbench (CLC Bio Qiagen) and lists of single nucleotide polymorphisms (SNPs) were generated. PCR primers were designed to amplify 500–700 bp sequences covering the putative SNP to verify the SNP (data not shown). The PCR products were purified with the QIAquick PCR purification kit (Qiagen GmbH) sent to Macrogen Europe and analyzed in the CLC Genomics Workbench (data not shown).

### Analytical methods

*Tryptophan analysis by GC*–*MS,* see Additional methods 2.

*Reverse transcription*-*PCR set*-*up and analysis,* see Additional methods 3.

## Results and discussion

### Obtaining natural tryptophan producing *Bacillus subtilis* strains

To obtain a Trp over-producing strain, different recombinant DNA techniques have been applied (Ikeda 2006) but, in the food and feed industry, recombinant DNA techniques are highly controversial. In this study, a natural approach has been applied to obtain *B. subtilis* strains with increased production levels of Trp by iterative rounds of mutagenesis and stepwise selection on analogues. Several analogues were tested and the best Trp producers consistently were obtained from the dominant selection of colonies able to grow on 5-FT and 8-AZA, as outlined in Supplementary Fig. 1 and Additional methods 1. A total of 370 candidates were assayed for Trp, 130 from WT1 and 240 from WT2. One Trp overproducing mutant was selected from each wild type; mutant 1 (M1) and mutant 2 (M2).

**Fig. 1:**
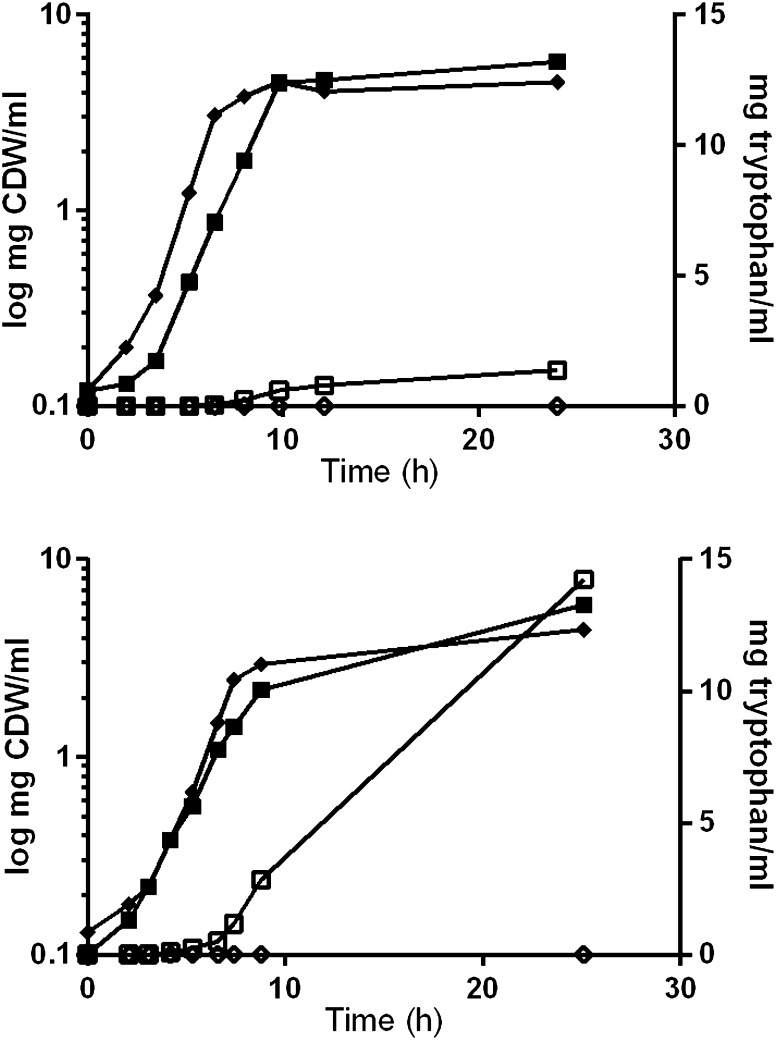
Cell dry weight (CDW) and tryptophan (Trp) production with the *B. subtilis* strains **a** wild type 1 (WT1) and mutant 1 (M1) and **b** wild type 2 (WT2) and mutant 2 (M2). Symbols OD WT (*filled diamond*), OD M (*filled square*), Trp WT (*open diamond*), Trp M (*open square*)

Growth and Trp production by the two wild type strains and their respective mutants were investigated in a defined minimal medium in aerobic batch fermentations (Fig. 1). The WT1 had 1.5 h shorter lag phase than M1, and maximal specific growth rates were 0.71 and 0.55 h^−1^, respectively. The WT2 and M2 had similar growth characteristics, but differentiate in maximal specific growth rate; 0.63 and 0.42 h^−1^, respectively. The maximal specific growth rate of the two WT strains used in the current study was higher than for the respective Trp mutants. Since there were only very few single nuclear polymorphisms (SNPs) introduced between the wild type strains and the mutant strains, it indicates that the slower growth rates is caused by the over-expression of the Trp operon.

Fitness of the strain is important in feed applications. Good growth and sporulation in production is crucial and the microorganism should survive and ideally proliferate in the pig intestine. Compared to previous investigations, where bacilli were developed for commercial Trp production, both M1 and M2 were sensitive to lower concentrations of 5-FT and 8-AZA (Kurahashi et al. 1987). The use of WT strains are not expected to harbor as many genetic changes as transductants or auxotrophs and would likely favor the overall fitness.

### Modifying tryptophan regulation

There were three SNPs difference between the WT1 and the M1 strains (Table 2). The most interesting difference between WT1 and M1 was the base deletion in the stop codon of the *mtrB* gene resulting in a frameshift and extension of the open reading frame with 34 amino acids (Supplementary Table 2). The *mtrB* gene encodes the TRAP protein which regulates tryptophan synthesis, and the expression of the *mtrB* gene is regulated by the amount of Trp. Between the WT2 and the M2 strains there were two SNPs difference (Table 2). The most interesting of the SNPs was located upstream the *trpS* gene, encoding tryptophanyl-tRNA synthase which is involved in tryptophan regulation.

**Table 2:** Location of nucleotide change between the two wild type strains and the corresponding mutants

Gene	Function	Nucleotide change in mutant 1	Nucleotide change in mutant 2	Amino acid change
*mtrB*	TRAP, tryptophan regulation	225a → deletion	n.a.	Abolishes stop codon
*ykvU*	Spore membrane protein	323t → c	n.a.	Ala108Val
*yflD*	Hypothetical protein	93a → g	n.a.	No amino acid change
*trpS*	15 bp upstreams the tryptophanyl-tRNA synthase start codon	n.a.	g → a	
*pyrH*	Uridine monophosphate kinase	n.a.	260c → 260a	Asp87Ala

*n.a.* not applicable

To investigate if the genes in the Trp operon were differently regulated, relative expression of the genes in the Trp operon was measured with reverse transcription (RT)-PCR (Table 3). The RT-PCR results showed that the genes in the *trpEDCFBA* operon were up-regulated 3 to 9 fold for M1 compared to WT1, whereas for M2, the increase was 13 to 30 fold.

**Table 3:** Tryptophan operon genes affected in the *B. subtilis* Trp mutant strains (M) compared to the wild type strains (WT)

Gene	Function	Gene expression ratio^a^	
		M1/WT1	M2/WT2
*trpA*	Tryptophan synthase alpha chain	2.8	12
*trpB*	Tryptophan synthase beta chain	4.1	15
*trpC*	Indole-3-glycerol phosphate synthase	4	20
*trpD*	Anthranilate phosphoribosyltransferase	4.2	26
*trpE*	Anthranilate synthase component 1	5.2	29
*trpF*	*N*-(5’-phosphoribosyl)anthranilate isomerase	9.2	20
*trpP*	Tryptophan transport protein	1.3	−1.1
*trpS*	Tryptophanyl-tRNA synthase	−2.9	−1.3
*pyrH*	UMP kinase	−1.2	1.3
*mtrB*	Regulating the trp operon	−1.9	−1.9
*ykvU*	Sporulation protein	−3.5	−21

^a^Primers were added at 300 nM. The primer efficiencies were in the range of 85–102%. The *gyrA* and *gyrB* genes were selected as multiple reference genes. PCR conditions: 20 s at 95 °C, followed by 40 cycles of 3 s of denaturation at 95 °C and 1 min of priming/elongation at 60 °C. The standard deviation of the RT-PCR results was up to 20%

The Trp operon is tightly regulated in *B. subtilis* (Sonenshein et al. 2002). The SNP located in the *mtrB* gene in M1 abolishes the stop-codon and could therefore generate a larger TRAP (Supplementary Table 2). If the eleven-subunit TRAP structure is different, it can be speculated that this extended protein could not bind the leader RNA sequence properly, Trp does not bind into the TRAP pocket or the multimeric protein complex cannot assembly into a functional complex. Subsequently, Trp synthesis would not be repressed at high Trp concentrations. The RT-PCR results indeed showed that the *trp* genes were up-regulated in M1. In literature, *B. subtilis* strains with modifications of the *mtrB* gene have shown increased expression of the genes in the Trp operon (Berka et al. 2003; Yu et al. 2014).

The *trpS* gene encodes tryptophanyl-tRNA synthase which in two steps catalyzes the attachment of Trp to its cognate tRNA. Results from a temperature sensitive *B. subtilis tprS* mutant suggested that *B. subtilis* has a mechanism for recognizing the accumulation of uncharged tRNA^Trp^ and respond to this by increasing the Trp biosynthetic genes (Lee et al. 1996; Steinberg and Anagnostopoulos 1971). Even though our RT-PCR indicates that the *trpS* gene expression is more or less unaffected in the mutant strains, small changes in expression could have a major impact. Increased levels of uncharged tRNA^Trp^ in *B. subtilis* induce synthesis of an anti-TRAP protein which antagonizes the TRAP function and thereby increases all genes regulated by TRAP (Valbuzzi et al. 2002). This could be the case for M2, which has a SNP up-stream the *trpS* gene and increased expression of the Trp operon. A mutation of the *trpS* gene in *B. subtilis* affects Trp metabolism and transcription in *B. subtilis* (Berka et al. 2003). The regulation of the *trpS* gene was modified, generating a difference in the uncharged tRNA^Trp^ levels. This could alter the anti-TRAP protein levels in the bacteria, and thereby increase the Trp synthesis genes. In the current experiment, M2 had 14 times higher Trp production than M1 and up to 6 times increased expression of the genes in the Trp pathway compared to M1.

*B. subtilis* strains with mutations in the *mtrB* and *trpS* increased the expression of the Trp operon and produced more Trp. Earlier reports also show importance of these genes for the Trp operon (Berka et al. 2003). However they report the *mtrB* gene to have the strongest impact of the expression of the Trp operon, but Trp production was not quantified and SNP´s were not identical.

### Applications in feed

*Bacillus* spores are used as probiotics for both animals and humans and has been used in the pig industry for 10–15 years, with positive effects on weight gain, reduced mortality rate and diarrhea (Alexopoulos et al. 2004). The feed is of crucial importance for both the farmers and the pigs. It is a major cost, and needs to be optimally composed for healthy fast-growing pigs. In intensive pig production areas, nitrogen pollution is also considered as a major problem. By supplementing crystalline amino acids to the animal feed, less dietary crude protein is used—which, due to reduced nitrogen excretion, beneficial for the environment. Thus the objective of the study was to develop probiotic *B. subtilis* strains producing Trp for use in situ. This add-on concept would reduce the feed cost by omitting or reducing the amount of crystalline amino acids which has to be added.

The effect of amino acid producing *Bacillus* in feed has only recently been investigated for a *Bacillus* strain producing Val (Nørgaard et al. 2012). In pigs fed a low Val diet including a Val producing *B. subtilis*, a tendency towards higher Val concentrations in mucosa from jejunum was observed but a growth performance similar to the sufficient diet was not obtained (Nørgaard et al. 2012). Pig trials were performed with M1 and M2 (Torres-Pitarch et al. 2016 JV Nørgaard, unpublished data). Addition of M1 at 10 and 100 times standard dose (which equals 1.28 × 10^6^ *Bacillus* spores/g feed) in the feed showed tendencies towards elevated Trp levels in the blood plasma, and a numerical, but not statistically significant, increase in feed intake and weight gain. The dose response experiment showed that a 100 times dose of M1 was equivalent to a dietary supplementation of 0.11 g Trp/kg feed, whereas a 10 times dose only supplied 0.02 g Trp/kg feed, measured as weight gain. In another study with M2 added to pig feed in a 10 times dose, no effect could be seen neither in Trp blood plasma concentrations nor in growth performance (Torres-Pitarch et al. 2016; JV Nørgaard, unpublished data). Interestingly, M1 with the lowest Trp production in vitro had the best results in situ, which might be related to the SNP in the germination gene *ykvU*. Neither M1 nor M2 were able to supply enough Trp to alleviate the dietary deficiency, when supplemented in the standard dose.

Mutant 1 improved protein utilization in the pig and the mode of action might be an increased Trp production by the mutant. This feature is useful and could reduce the protein supply to the pig and thereby improve the farmer’s economy and reduce nitrogen pollution. However, the amount of improved protein utilization needs to be increased before it will create value for the feed industry. A GMO solution could be one way of going forward but it is questionable if the European consumers are ready for this solution. Another option could be the ongoing process of natural mutation as used in this paper but the understanding of the relation between in vitro and in vivo results needs to be improved before a valuable solution might be useful for the feed industry.

## Electronic supplementary material

Below is the link to the electronic supplementary material.

Supplementary material 1 (DOCX 12 kb)

Supplementary material 2 (DOCX 12 kb)

Supplementary material 3 (DOCX 45 kb)

Supplementary material 4 (DOCX 13 kb)

Supplementary material 5 (DOCX 13 kb)

Supplementary material 6 (DOCX 12 kb)
