# Supplementary material for: Development of Bacillus subtilis mutants to produce tryptophan in pigs
Source: Biotechnol Lett. 2016 Nov 3;39(2):289–95. doi: 10.1007/s10529-016-2245-6 (PMC5247549; doi:10.1007/s10529-016-2245-6)
Supplement: Supplementary file 1 — Supplementary material 1 (DOCX 12 kb) [file 10529_2016_2245_MOESM1_ESM.docx]

**Additional methods 2** Tryptophan analysis by GC-MS

The amino acids in the samples were analyzed as methyl chloroformate derivates by GC-MS. Equal amounts (150 µl) of sample and internal standard norvaline was mixed with 200 µl 1-methanol/pyridine 32/8 % (v/v). Then 25 µl methyl chloroformate (MCF) was added, after mixing 500 µl 1 % MCF/CHCl_3_ (v/v) was added and mixed vigorously until phase separation occurred. After centrifugation 2 µl sample was injected in split mode at 350 °C onto a DB-XLB column (15 m x 0.25 mm x 0.25 µm, Agilent 122-1212), with helium as the carrier gas at a total flow of 26.5 ml/minute. The oven program was 110 °C initially, increasing by 20 °C/min. up to 320 °C. Peaks were identified by MSD in SIM mode, up to four ions were collected in each group. For quantification ion extracts were used.

Development of *Bacillus* *subtilis* mutants to produce tryptophan in pigs. Biotechnology Letters. Karin Bjerre, Mette D. Cantor, Jan V. Nørgaard, Hanne D. Poulsen, Karoline Blaabjerg, Nuria Canibe, Bent B. Jensen, Birgitte Stuer-Lauridsen, Bea Nielsen, Patrick M.F. Derkx. Chr. Hansen A/S, Bøge Allé 10-12, DK-2970 Hoersholm, Denmark, dkkbj@chr-hansen.com
