# Supplementary material for: Development of Bacillus subtilis mutants to produce tryptophan in pigs
Source: Biotechnol Lett. 2016 Nov 3;39(2):289–95. doi: 10.1007/s10529-016-2245-6 (PMC5247549; doi:10.1007/s10529-016-2245-6)
Supplement: Supplementary file 2 — Supplementary material 2 (DOCX 12 kb) [file 10529_2016_2245_MOESM2_ESM.docx]

**Additional methods 3.** *Reverse transciption-PCR set-up and analysis*

The RT-PCR was designed with the two-step method; RNA was purified, cDNA was generated and PCR reactions were carried out with the cDNA material. Cells were harvested in the exponential phase at OD 2. The cells were treated with RNAprotect Bacteria Reagent (Qiagen GmbH). Total RNA was purified by the RNeasy kit (Qiagen GmbH). The quantity and quality of the RNA was measured by an Agilent 2100 Bioanalyser (Agilent Technologies Denmark ApS). cDNA was prepared from total RNA with high capacity RNA-to-cDNA kit with hexamer priming (Applied Biosystems).

Primers for the RT-PCR´s were designed by the Universal probe library assay design center online with 60 °C as annealing temperature in all cases (Roche Diagnostics GmbH). The unique DNA sequences from the *B. subtilis* strains in this study were used. The comparative RT-PCR experiments were performed from two biological replicates with four technical replicates each with the FAST SYBR Green Master Mix (Applied Biosystems) on a 7500 Fast Real-Time PRC system machine (Applied Biosystems). The RT-PCR data was analyzed with the 7500 software v.2.0.5 (Applied Biosystems).

Development of *Bacillus* *subtilis* mutants to produce tryptophan in pigs. Biotechnology Letters. Karin Bjerre, Mette D. Cantor, Jan V. Nørgaard, Hanne D. Poulsen, Karoline Blaabjerg, Nuria Canibe, Bent B. Jensen, Birgitte Stuer-Lauridsen, Bea Nielsen, Patrick M.F. Derkx. Chr. Hansen A/S, Bøge Allé 10-12, DK-2970 Hoersholm, Denmark, dkkbj@chr-hansen.com
