# Supplementary material for: Development of Bacillus subtilis mutants to produce tryptophan in pigs
Source: Biotechnol Lett. 2016 Nov 3;39(2):289–95. doi: 10.1007/s10529-016-2245-6 (PMC5247549; doi:10.1007/s10529-016-2245-6)
Supplement: Supplementary file 3 — Supplementary material 3 (DOCX 45 kb) [file 10529_2016_2245_MOESM3_ESM.docx]

**Supplementary Figure 1** Schematic flow of generation of tryptophan (Trp) producing strains. **a** wild type 1 (WT1) to mutant 1 (M1) and **b** wild type 2 (WT2) to mutant 2 (M2). The two different wild types (WT) were exposed to UV irradiation for different times and the UV pools thus obtained propagated overnight in a non-selective medium. The UV pool was then spread on the different analogues in different inhibitory concentrations and the fastest growing colonies picked for Trp assay. The best Trp producer was then subjected to a new round of UV and/or dominant selection, in an iterative process. Abbreviations: 5-fluoro-DL-tryptophan (5-FT) and 8-aza-guanine (8-AZA)

**
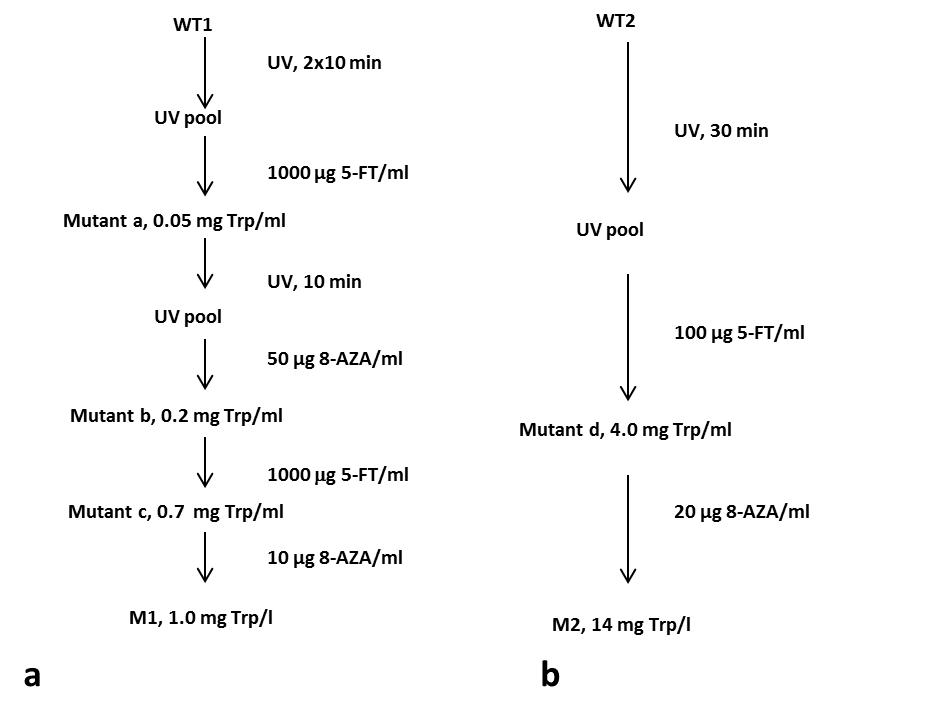
**

Development of *Bacillus* *subtilis* mutants to produce tryptophan in pigs. Biotechnology Letters. Karin Bjerre, Mette D. Cantor, Jan V. Nørgaard, Hanne D. Poulsen, Karoline Blaabjerg, Nuria Canibe, Bent B. Jensen, Birgitte Stuer-Lauridsen, Bea Nielsen, Patrick M.F. Derkx.

Chr. Hansen A/S, Bøge Allé 10-12, DK-2970 Hoersholm, Denmark, dkkbj@chr-hansen.com
