# Supplementary material for: Development of Bacillus subtilis mutants to produce tryptophan in pigs
Source: Biotechnol Lett. 2016 Nov 3;39(2):289–95. doi: 10.1007/s10529-016-2245-6 (PMC5247549; doi:10.1007/s10529-016-2245-6)
Supplement: Supplementary file 4 — Supplementary material 4 (DOCX 13 kb) [file 10529_2016_2245_MOESM4_ESM.docx]

**Supplementary Table 1** Composition of chemically defined medium (CDM)

| CDM1^a^ | | CDM2^b,c^ | |
| --- | --- | --- | --- |
| Chemical | Amount (g/l) | Chemical | Amount (g/l) |
| (NH4)2SO4 | 1 | NH4Cl | 0.50 |
| KH2PO4 | 8.6 | KH2PO4 | 9.52 |
| MgSO4.7H2O | 0.2 | MgCl.6H2O | 2.46 |
| FeSO4.7H2O | 0.01 | FeCl_3_.6H2O | 0.00108 |
| MnSO4.4H2O | 0.01 | MnCl2.4H2O | 0.079 |
| Sodium citrate | 0.5 | Sodium citrate | 1 |
| Glucose | 5 | Na_2_SO_4_ | 0.142 |
| Water | 1000 | NaNO_3_ | 0.085 |
| pH adjusted to | 6.5 | KCl | 0.075 |
|  |  | CaCl_2_ | 0.111 |
|  |  | Glucose | 5 |
|  |  | Water | 1000 |
|  |  | pH adjusted to | 6.5 |

^a^ (Leitch et al. 1996)

^b^ (Kurahashi et al. 1985)

^c^ For CDM2 the various medium components were dissolved in 800 ml of distilled water, pH adjusted to pH 6.5 by the addition of either hydrochloric acid or sodium hydroxide and the volume adjusted to 1000 ml. The medium was filter sterilized under vacuum through a 0.22 µm membrane.

Development of *Bacillus* *subtilis* mutants to produce tryptophan in pigs. Biotechnology Letters. Karin Bjerre, Mette D. Cantor, Jan V. Nørgaard, Hanne D. Poulsen, Karoline Blaabjerg, Nuria Canibe, Bent B. Jensen, Birgitte Stuer-Lauridsen, Bea Nielsen, Patrick M.F. Derkx. Chr. Hansen A/S, Bøge Allé 10-12, DK-2970 Hoersholm, Denmark, dkkbj@chr-hansen.com
