# Supplementary material for: Development of Bacillus subtilis mutants to produce tryptophan in pigs
Source: Biotechnol Lett. 2016 Nov 3;39(2):289–95. doi: 10.1007/s10529-016-2245-6 (PMC5247549; doi:10.1007/s10529-016-2245-6)
Supplement: Supplementary file 5 — Supplementary material 5 (DOCX 13 kb) [file 10529_2016_2245_MOESM5_ESM.docx]

**Supplementary Table 2** The TRAP amino acid sequence in wild type 1 and the putative new TRAP amino acid sequence in mutant 1

| Strain | Amino acid sequence |
| --- | --- |
| Wild type 1 | Met N Q K H S S D F V V I K A V E D G V N V I G L T R G T D T K F H H S E K L D K G E V I I A Q F T E H T S A I K V R G E A L I Q T A Y G E M K S E K K Stop |
| Mutant 1^a^ | Met N Q K H S S D F V V I K A V E D G V N V I G L T R G T D T K F H H S E K L D K G E V I I A Q F T E H T S A I K V R G E A L I Q T A Y G E M K S E K K **I S W L S P L L K A N A R K Q R E D T A N F F F L C Y N D T C V S E** Stop |

^a^ The additional amino acids in bold.

Development of *Bacillus* *subtilis* mutants to produce tryptophan in pigs. Biotechnology Letters. Karin Bjerre, Mette D. Cantor, Jan V. Nørgaard, Hanne D. Poulsen, Karoline Blaabjerg, Nuria Canibe, Bent B. Jensen, Birgitte Stuer-Lauridsen, Bea Nielsen, Patrick M.F. Derkx. Chr. Hansen A/S, Bøge Allé 10-12, DK-2970 Hoersholm, Denmark, dkkbj@chr-hansen.com
