# Supplementary material for: Development of Bacillus subtilis mutants to produce tryptophan in pigs
Source: Biotechnol Lett. 2016 Nov 3;39(2):289–95. doi: 10.1007/s10529-016-2245-6 (PMC5247549; doi:10.1007/s10529-016-2245-6)
Supplement: Supplementary file 6 — Supplementary material 6 (DOCX 12 kb) [file 10529_2016_2245_MOESM6_ESM.docx]

**Additional methods 1** UV mutagenesis and dominant selection protocols

*UV mutagenesis*

The wild type *B. subtilis* strains were inoculated directly from glycerol stock with 3.5 ml into 25 ml VIB and incubated at 37 °C and 150 rpm, to optical density (OD) at 600 nm between 0.4–0.9. The exponentially growing cells were subjected to UV-irradiation with 4 ml of undiluted culture in an open Petri dish. UV exposure was in a UV-Crosslinker (Amersham Life Science) at the highest effect possible, 70 mJ/cm2. Different sets of treatments were carried out, up to a total of 40 min, in 2-3 periods to avoid heating the bacteria excessively. After each period the culture was mixed by swirling the petri dish. For measuring viability following the UV treatment 1 ml of the cells (UV pools) was immediately mixed with 5 ml VIB, the tubes wrapped in aluminum foil and incubated over night at 37 °C and 150 rpm. The viability of the cells after the UV treatments and of an untreated control was determined by plating 10-fold dilutions on VIB agar. The plates were wrapped in aluminum foil and incubated at 37 °C for 24 h before counting. UV pools where the number of viable cells was reduced to 1-5 % were used for the dominant selection of Trp overproducing mutants.

*Dominant selection*

For dominant selection, OD adjusted culture was spread with a needle on the surface of CDM1 agar added the desired type and concentration of analogue. After incubation at 37 °C, with daily inspection, colonies were picked, grown over night in VIB and frozen as glycerol stocks.

Development of *Bacillus* *subtilis* mutants to produce tryptophan in pigs. Biotechnology Letters. Karin Bjerre, Mette D. Cantor, Jan V. Nørgaard, Hanne D. Poulsen, Karoline Blaabjerg, Nuria Canibe, Bent B. Jensen, Birgitte Stuer-Lauridsen, Bea Nielsen, Patrick M.F. Derkx. Chr. Hansen A/S, Bøge Allé 10-12, DK-2970 Hoersholm, Denmark, dkkbj@chr-hansen.com
